# Chronic Conditions: Beckett, Bergson and Samuel Johnson

**DOI:** 10.1007/s10912-015-9372-2

**Published:** 2016-01-09

**Authors:** Ulrika Maude

**Affiliations:** Department of English, University of Bristol, 3/5 Woodland Road, Clifton, Bristol, BS8 1TB UK

**Keywords:** Samuel Beckett, Henri Bergson, Samuel Johnson, Mechanisation, Intentionality, Language, Neurology, Tourette's syndrome

## Abstract

This article analyses the work of the twentieth-century late modernist Samuel Beckett, in light of the turn-of-the-century anti-rationalist Henri Bergson (1859-1941) and the eighteenth-century neoclassicist Samuel Johnson (1709-1784). What unites these three very different thinkers is a concern over habitual, automatic and involuntary behavior, which in all three cases has a distinctly neurological dimension. Beckett’s writing explores the Bergsonian notion, informed by medicine and experimental psychology, of the limitations of agency, of “the deep-seated recalcitrance of matter,” and of the human as always already inflicted by the mechanical, a fact that is poignantly highlighted by the case of Samuel Johnson. Through his encounter with Johnson, Beckett registers a paradigm shift in the understanding of subjectivity. Whereas Bergson aims, throughout his career, to *contest* the mechanical, habitual and automatic that threaten to encrust themselves upon the living, in Beckett’s often uncannily Johnsonian writing, the habitual and the automatic become progressively more central, until in the late works, habit and mechanical behavior constitute a tenuous, fraught and primitive ontology, the residues of an agential self.

This article analyses the work of the twentieth-century late modernist Samuel Beckett, in light of the turn-of-the-century anti-rationalist Henri Bergson (1859-1941) and the eighteenth-century neoclassicist Samuel Johnson (1709-1784). What unites these three very different thinkers, I will argue, is a concern over habitual, automatic and involuntary behaviour, which in all three cases has a distinctly neurological dimension.

Samuel Beckett’s interest in the work of the French philosopher, Henri Bergson, began in or around 1930 when he read *Laughter: An Essay on the Meaning of the Comic*, first published in 1899. Beckett had recently returned from a two-year period (1928 to 1930) as *Lecteur* at the École Normale Supérieure where he would have had ample exposure to Bergson’s ideas.^1^ It is unclear whether Beckett would have felt compelled to read Bergson during his residence in Paris, for Bergsonism was just beginning to wane in the period of Beckett’s appointment at the École Normale (See Addyman 2012). French literary criticism, however, was predominantly Bergsonian in outlook, and during his brief period as Lecturer in French at Trinity College Dublin, from 1930 to 1931, Beckett made ample references to Bergson in his undergraduate lectures on French literature, for instance distinguishing ‘between Bergson’s conception of time and Proust’s [,] and between Bergson’s attitude to language and Gide’s’ (Pilling 1997, 237), as the lecture notes of Beckett’s former student, Rachel Burrows, in Trinity College Dublin archives reveal. Furthermore, in October 1930, Beckett wrote to his publisher, Charles Prentice, informing him that he wanted to add some pages to his book on Proust*,* published in 1931, ‘in part to separate Proust’s intuitivism from Bergson’s’ (Pilling 1997, 237).^2^ Beckett’s reading notes on Bergson have not been recovered, but I will argue that Bergson’s thinking did have a lasting influence on Beckett’s work. His publisher, John Calder, recalls conversations with Beckett about Bergson from the mid-1950s until Beckett’s death in 1989 that Calder says, “went on all night” (2001, 109).

Bergson’s extended essay, *Laughter,* was substantially influenced by medical discoveries and especially the performative and often spectacular culture of late-nineteenth-century neurology. In 1862, Jean-Martin Charcot had been appointed head physician at the Salpêtrière Hospital in Paris. Under his directorship, the hospital underwent numerous reforms, including the laicization of its nursing staff, an increase in the number of beds, “better salaries for ancillary staff, improved bathing facilities, as well as laboratories, a museum, a new lecture hall” and, from 1882, a *Service des hommes* (Harris 1991, xix). By the second half of the 1870s, Charcot’s famous Tuesday lectures, which attracted the public in extraordinary numbers, were a central feature of “Parisian intellectual life” (Harris 1991, xviii). At the lectures, Charcot exhibited his patients and developed his case studies before admiring crowds. In 1882, he inaugurated the first neurology clinic in Europe. At his clinic, Charcot and his many eminent students made important discoveries in the understanding of such conditions of the nervous system as Parkinson’s disease, epilepsy, Tourette’s syndrome and hysteria. These were brought to public attention at the Tuesday lectures and in the many journals founded in the period of the Third Republic such as *Le Progrès médical,* in which Charcot published a number of his lectures. *La Nouvelle iconographie de la Salpêtrière,* the hospital’s own publication, distributed images of epileptics, hysterics and sufferers of other neurological conditions; it ran from 1888 to 1918 and had George Gilles de la Tourette as one of its founders*.* Knowledge of Charcot’s discoveries entered even the popular newspapers and magazines, and a number of Charcot’s patients became celebrities in their own right. So pervasive was Charcot’s work and so profound was its impact on the popular imagination that it rapidly influenced the performance style of the Parisian cabaret and vaudeville “with a new repertoire of movements, grimaces, tics and gestures” (Gordon 2004, 93), which mimicked the comportment and disposition of the Salpêtrière patients. Comedians in particular sported convulsive and marionette-like gaits and movements, and mime troupes and singers followed suit in performances that seemed to cast doubt over received notions of the body’s functioning and by implication, the wider questions of agency and free will. Many cabaret and music hall performers went on to have successful careers in silent film, which, as a genre, adopted the frenetic, convulsive and automatic performance style of vaudeville and cabaret. As Rae Beth Gordon writes: “There is a continuous line and directing force running from the cabaret and café-concert performances of the last quarter of the nineteenth century, through the films of Méliès and the musicals of Ernst Lubitsch […].The uniting element is hysterical gesture and gait” (2004, 111). Bergson’s *Le Rire* was itself centred around the notion of “automatic gesture and word,” and one can trace a direct genealogy between Charcot’s work, the popular culture of the period, and Bergson’s theory of comedy. So fashionable and intriguing did hysteria and neurological disorders prove around the turn of the century that they generated, besides a new performance style, a number of songs and literary works such as Guy de Maupassant’s short story, “Le Tic” (“The Spasm”) from 1884 or T. S. Eliot’s poem “Hysteria” from 1915.

Bergson’s *Laughter* was anxiously indebted to neurological discoveries and especially the dyskinesia and the various automatisms that presented in neurological disorders and that figured so prominently in the performance culture of the period. Bergson argued in his book that “The attitudes, gestures and movements of the human body are laughable in exact proportion as that body reminds us of a mere machine” (1911, 29). Humour, Bergson reiterated, arises from “Something mechanical encrusted on the living” (37), for “a comic character is generally comic in proportion to his ignorance of himself” (16). This makes the subject appear as if deprived of his or her essential freedom:

The soul imparts a portion of its winged lightness to the body it animates: the immateriality which thus passes into matter is what is called gracefulness. Matter, however, is obstinate and resists. It draws to itself the ever-alert activity of this higher principle, would fain convert it to its own inertia and cause it to revert to mere automatism. It would fain immobilise the intelligently varied movements of the body in stupidly contracted grooves, stereotype in permanent grimaces the fleeting expressions of the face, in short imprint on the whole person such an attitude as to make it appear immersed and absorbed in the materiality of some mechanical occupation instead of ceaselessly renewing its vitality by keeping in touch with a living ideal. Where matter thus succeeds in dulling the outward life of the soul, in petrifying its movements and thwarting its gracefulness, it achieves, at the expense of the body, an effect that is comic. (28-29)

What neurological conditions such Parkinson’s disease, Tourette’s syndrome and epilepsy had in common and what was seen as a source of black humour in cabaret and early cinema was the body’s seemingly-mechanical capacity to act outside of the realm of conscious control. Neurological disorders which informed the performance style of music hall, vaudeville, cabaret and film, and as a consequence, Bergson’s work, questioned notions of agency and intentionality and hence cast serious doubt over received notions of subjectivity, suggesting that the mechanical, the automatic and the involuntary were in fact integral to the self. These pathologies “raised serious questions about the philosophical viability of the doctrine of free will” (Harris 1991, xvii), something that Charcot and his students suggested was a mere metaphysical construct. Of relevance here was also Charcot and his students’ anti-clericalism, which permeated not only their political views but also their research (Harris 1991, xix).

The question of free will is a central concern in Bergson’s work, as the title of his doctoral thesis, and his first published book, *Time and Free Will: An Essay on the Immediate Data of Consciousness,* from 1889, reveals. It is crucial, for instance, to his notion of duration (*durée*), whose flow is constantly threatened by habit, repetition and automatism. Bergson in fact appears determined to defend the faculty of free will to the point where his work frequently unveils a deep-rooted anxiety over its limitations. In his essay, *Laughter,* habitual, mechanical, ossified comportment, as we have seen, appears as a source of humour, but it is also a locus of intense anxiety, which persists throughout Bergson’s writing. In the essay, Bergson conceptualises laughter as “a bursting out of life and elasticity in the face of the intolerable stiffening of life into automatic or repeated gestures” (Connor 2008). As Bergson puts it, “rigidity is the comic, and laughter is its corrective” (1911, 21). However, as he often acknowledges, laughter itself can function mechanically, as an involuntary somatic reaction beyond intentional control. Steven Connor writes:

…in laughing at what is inhumanly inelastic, we actually mirror the condition that is said to be comic. This confusion between stimulus and response, or between the laughable and the laugh, runs through Bergson’s account. “[I]nvoluntarily I laugh,” he writes (Bergson 1911, 32); and when he asserts that “it is really a kind of automatism that makes us laugh [une espèce d’automatisme qui nous fait rire],” it is not certain whether he means that we laugh at automatism or that we laugh as an instance of it. (2008, n.p.)

Certain aspects of Beckett’s humour, as well as his attitude to repetition and compulsion owe much to his reading of Bergson’s work. Although Beckett rejects Bergson’s Cartesian division into spirit and matter, his influence can be said to be pervasive, for it is not merely Beckett’s humorous works that are infused by Bergsonian ideas. The humour, after all, begins to recede from Beckett’s writing after *Happy Days* (1961), but his work nonetheless retains his interest in the mechanized and ossified structures of comportment as late plays such as *Footfalls* (1975), *Rockaby* (1980), *Quad* (1982) and *What Where* (1983) so clearly attest. In these plays, Beckett’s characters “sometimes seem to be losing species, regressing to the subhuman, trying to rehearse the figures of instinct but botching the job,” as Daniel Abright has put it (2003, 69).

What is perhaps most pertinent to Beckett’s work, however, is Bergson’s attitude to language. For the ossified and rigid habits that everywhere threaten to stunt vitality – the *élan vital* so central to Bergson’s thinking – are also lurking at the very heart of language itself. Bergson writes of language that,

We feel it contains some living element of our own life; and if this life of language were complete and perfect, if there were nothing stereotype in it, if, in short, language were an absolutely unified organism incapable of being split up into independent organisms, it would evade the comic as would a soul whose life was one harmonious whole, unruffled as the calm surface of a peaceful lake. There is no pool, however, which has not some dead leaves floating on its surface, no human soul upon which there do not settle habits that make it rigid against itself by making it rigid against others, no language, in short, so subtle and instinct with life, so fully alert in each of its parts as to eliminate the ready-made and oppose the mechanical operations of inversion, transposition, etc., which one would fain perform upon it as on some lifeless thing. (1911, 129-30)

The machinic, rigid and automatic qualities of language, in other words, haunt Bergson’s oeuvre, and the assumption that language might function mechanically becomes progressively more pronounced in his thinking. In *Creative Evolution* (1998), Bergson makes the even bolder suggestion that “The most living thought becomes frigid in the formula that expresses it. The word turns against the idea. The letter kills the spirit” (127).

In 1931, shortly after reading Bergson, Beckett turned his attention to Max Nordau’s *Degeneration* (*Entartung,* 1892), which he read in the 1895 English translation. Nordau, a doctor of Hungarian origin, had himself – upon his arrival in Paris in 1880 – undertaken medical research under the tutelage of Jean-Martin Charcot and after defending his thesis in 1882, had remained closely involved with the Charcot circle (Gambor 2009, 138). Beckett’s ‘*Dream* Notebook’, now held in the University of Reading Beckett Archive, contains his reading notes on Nordau’s book. Amongst them is a quotation from Spinoza that Beckett copied from Nordau: “If a stone flung by a human hand could think it would certainly imagine that it flew because it wished to fly” (1999, 89). Nordau goes on to elaborate: “Many mental conditions and operations of which we become conscious are the result of causes which do not reach our consciousness. In this case we fabricate causes *a posteriori* for them, satisfying our mental need of distinct causality, and we have no trouble of persuading ourselves that we have now truly explained them” (1993, 20). Although Nordau’s thesis expresses an anxiety about social, cultural and biological degeneracy, his observation is pertinent to the findings of nineteenth-century neurology, which he frequently references, as well as to the problematisation of free will by Charcot and his many eminent followers. One of the neurological conditions Nordau references is Tourette’s syndrome, as Beckett’s notes record. His notebook registers two of the symptoms of the syndrome: “Echolalia (word & sound repetition)” and “corprolalia (mucktalk)” (Beckett 1999, 91, 97). Beckett read the following passage in Nordau’s book: “Gilles de la Tourette has coined the word ‘corprolalia’ (mucktalk) for obsessional explosions of blasphemies and obscenities which characterise a malady described most exhaustively by M. Catrou, and called by him ‘disease of convulsive tics’” (1993, 499). Nordau, who repeatedly attacks Émile Zola in the book, goes on to say that “M. Zola is affected by coprolalia to a very high degree” (1993, 499).^3^ Although Nordau’s claim about Tourette’s as an example of degeneration is deeply objectionable, his book does repeatedly draw attention to the close proximities between poetic language and language pathology, which would no doubt have captured Beckett’s imagination. The coprolalia of Tourette’s syndrome, after all, lends force to those qualities of language that escape conceptual definition, such as “volume, timing, tone, rhythm, emphasis, and patterns of sound repetition” (Brown and Kushner 2001, 550). For this reason, cursing not only embodies language, it enters “the realm of play and the nonreferential, which is also the realm of poetry, nonsense, and comedy” (Brown and Kushner 2001, 550). In Tourette’s, as in poetic language, it is the rhythms, rhymes, puns, sound qualities and polysemy of language that are foregrounded.

Although Tourette’s syndrome now bears the name of Georges Gilles de la Tourette, he was not the first nineteenth-century physician to publish on the seemingly-disconnected and diverse symptoms of the syndrome. In 1847, Ernest Billod had published his “Maladies de la Volonté” which, as the title suggests, saw the condition as a pathology of the will. In 1883, a book by the same title was published by Theodule Ribot, who described the symptoms of the Parisian Marquise de Dampierre – who suffered from Tourettic motor and verbal tics – as a case of “the idiocy of the will” (1883, 55; Eagle 2014a, 133). The condition that in 1885 came to be known as *maladie de Gilles de la Tourette* was, in other words, from its early conceptualisation, seen as a disorder of the will. In this new understanding and following Paul Broca’s localisation of speech in 1861, language, the supreme marker of agency and intentionality, proved at least in part to be mechanistic and self-generating. More recent neurological research has indeed suggested that Tourette’s is a disorder of “the very highest parts of ‘old brain’: the thalamus, hypothalamus, limbic system and amygdala, where the basic affective and instinctual determinants of personality are lodged” (Sacks 1985, 90). This also implies that certain functions of language are “overlaid on sensorymotor systems” that evolved to control motor functions and affect and that continue to do so now (Lieberman 2000, 1). The obscenities of Tourette’s syndrome function as “material articulations of language that gather up the power and emotions of seeming subcortical primal cries within discourses,” for obscenities are associated with “more or less automatic emotional responses” (Schleifer 2001, 575). Tourette’s as a disorder problematizes the distinction between voluntary and involuntary action, for like peristalsis, Tourettic tics can be suspended but not suppressed altogether, as Oliver Sacks (1985) has shown in his essay, “Witty Ticcy Ray”. In the phonic and verbal tics and utterances of Tourette’s, Brown and Kushner argue, the body itself becomes “a set of physiological impulses that are at once aberrant and self-seeking, expressive of the speaker’s circumstances while escaping his intentions” (2001, 538).

Another equally decisive factor in Beckett’s awareness of language pathologies, however, came a few years later and originated not in nineteenth-century but in eighteenth-century medical discourse. From 1936 to 1937, Beckett intensively researched the life of Samuel Johnson for a play he intended to write about the great eighteenth-century lexicographer. In a period of just over a year and mostly in the National Library of Dublin, Beckett read more than a dozen books about Dr. Johnson for his prospective but never completed play, "Human Wishes". Beckett’s play took its title from one of Johnson’s most acclaimed works, *The Vanity of Human Wishes* (1749), a poem which, like much of Beckett’s own work, might be characterised as a dramatisation of failure. Beckett’s fascination with Johnson is evinced by the three substantial exercise books that he filled with notes on his reading from this period.

Beckett’s interest in Johnson seems to have begun even earlier, in fact, for in a letter of 8 September 1934, he makes a reference to “the Lexicographer kicking the stone” (2009b, 223) – a story reported in James Boswell’s famous biography of Johnson “striking his foot with a mighty force against a large stone” in an attempt to refute Bishop Berkeley’s thesis of “the non-existence of matter, and that every thing in the universe is merely ideal” (1980, 471). Beckett also quotes a number of Johnson’s books in his “*Whoroscope* Notebook”, which dates from the 1930s, and at his death, Beckett owned a copy of the eighth edition (1799) of Johnson’s *Dictionary of the English Language*, which he had acquired during his formative years in Dublin (Smith 2002, 111). He consulted the dictionary regularly and was often bemused by Johnson’s definitions. In fact, according to Dirk Van Hulle and Mark Nixon, “the largest number of books in Beckett’s library” at the time of his death was “dedicated to the work of Samuel Johnson” (2013, 32). Beckett’s library also contained copies of three of the most important books he consulted in 1936-1937, namely James Boswell’s *Life of Johnson*, Mrs. Thrale’s *Anecdotes* and C. E. Vulliamy’s *Mrs. Thrale of Streatham*, and the fact that he purchased the Boswell and Thrale volumes in the 1960s and 1980s respectively, attests to his enduring interest in the life of Samuel Johnson (Smith 2002, 112).

Beckett’s original idea for his “Johnson blasphemy” – as he called his prospective play – focused on the unconsummated relationship between Johnson and Mrs. Thrale. His first “Human Wishes Notebook” is faithful to this idea although it tellingly contains far more notes on Johnson than on Mrs. Thrale. By the second notebook, however, Beckett’s interest has shifted from the relationship to Johnson himself with whom Beckett clearly identified and empathised. Frederik Smith has pointed out that “the figure of the declining Johnson became for him a sort of metaphor of Western man, academic and witty, alone, afraid of dying and yet intrigued by his own physical deterioration” (2002, 111). Indeed, in his second “Human Wishes Notebook,” Beckett takes nine pages of notes on Johnson’s medical conditions including his dropsy and its many symptoms, his asthma, his endocrine disorder, his depression (melancholy) and the attack of aphasia Johnson suffered at the age of seventy-three, after a minor stroke in the middle of the night on 17 June 1783 (Beckett 1936-1937). According to Mrs Thrale, Johnson composed a prayer in Latin immediately after the stroke in order “to satisfy himself that his mental powers remained unimpaired, and to keep them in exercise that they might not perish by permitted stagnation” (Boswell 1835, 232) – something Beckett’s notes also record. In the morning, Johnson wrote to his physician, Dr. Heberden: “It has pleased God by a paralytic stroke in the night to deprive me of speech” (Johnson 1992-1994, 149). While suffering from aphasia, Johnson continued to write letters but had no power of vocalisation. From one of the letters, we know that Johnson perceived his aphasia to derive from a problem of “the organs of speech” rather than from the brain. Through a close analysis of the nineteen letters written by Johnson after his stroke, Macdonald Critchley has concluded that Johnson suffered from dysphasia with symptoms of dysarthria as well and that his speech disorder was most probably Broca’s aphasia (aphemia) (1962, 35).

Tellingly, Beckett also took copious notes on Johnson’s nervous disorders, jotting down from Boswell’s biography that “’Such was the heat and irritability of his blood, that not only did he pare his hands to the quick, but scraped the joints of his fingers with a pen knife [sic], till they seemed quite red & raw”’ (Beckett 1936-1937, cited in Maude 2015). Most crucially, however, Beckett writes that “Miss Lucy Porter [Johnson’s stepdaughter] mentions his ‘convulsive starts & odd gesticulations’ + Fanny Burney” (Beckett 1936-1937, cited in Maude 2015). Quoting from Fanny Burney’s *Letters and Diaries*, Beckett notes:

In 1777: His mouth is almost constantly opening and shutting as if he were chewing. He has a strange method of frequently twirling his fingers & twisting his hands. His body is in constant agitation, see-sawing up and down; his feet are never a moment quiet, +, in short, his whole person is in perpetual motion (Beckett 1936-1937, cited in Maude 2015).

Boswell suggests in his biography that Johnson’s odd gesticulations appeared “to be of the convulsive kind, and of the nature of that distemper called St. Vitus’s dance,” affirming that “in this opinion I am confirmed by the description which Sydenham gives of that disease” (1980, 105). Beckett makes a note of this passage although as a number of twentieth-century commentators have pointed out, the symptoms of St. Vitus’s dance (Sydenham’s chorea) are temporary, and since Johnson suffered from motor and verbal tics all his life, his symptoms are unlikely to have been caused by the condition. Beckett would also have read in Boswell’s biography a rather striking passage focusing on the motor and vocal tics for which Johnson was famous:

…while talking or even musing as he sat in his chair, he commonly held his head to one side towards his right shoulder, and shook it in a tremulous manner, moving his body backwards and forwards, and rubbing his left knee in the same direction, with the palm of his hand. In the intervals of articulating he made various sounds with his mouth, sometimes as if ruminating, or what is called chewing the cud, sometimes giving a half whistle, sometimes making his tongue play backwards from the roof of his mouth, as if clucking like a hen, and sometimes protruding it against his upper gums in front, as if pronouncing quickly under his breath, *too, too, too*: all this accompanied sometimes with a thoughtful look, but more frequently with a smile. Generally when he had concluded a period, in the course of a dispute, by which time he was a good deal exhausted by violence and vociferation, he used to blow out his breath like a Whale. (Boswell 1980, 343).

We also know that Beckett read Birkbeck Hill’s *Johnsonian Miscellanies* in 1937. In it, he would have encountered Miss Francis Reynolds’ recollections of Johnson’s “extraordinary gestures,” of “his head, his hands and his feet often in motion at the same time” (Hill 1897, 274). Reynolds’ description is worth citing at some length. She recalls

…his extraordinary gestures or anticks with his hands and feet, particularly when passing over the threshold of a Door, or rather before he would venture to pass through *any* doorway. On entering Sir Joshua’s house with poor Mrs. Williams, a blind lady who lived with him, he would quit her hand, or else whirl about on the steps as he whirled and twisted about to perform his gesticulations; and as soon as he had finish’d, he would give a sudden spring, and make such an extensive stride over the threshold, as if he was trying for a wager how far he could stride, Mrs. Williams standing groping about outside the door, unless the servant or the mistress of the House more commonly took hold of her hand to conduct her in, leaving Dr. Johnson to perform at the Parlour Door much the same exercise all over again.

But the strange positions in which he would place his feet (generally I think before he began his straddles, as if necessarily preparatory) are scarcely credible. Sometimes he would make the back part of his heels to touch, sometimes the extremity of his toes, as if endeavouring to form a triangle, or some geometrical figure, and as for his gestures with his hands, they were equally as strange; sometimes he would hold them up with some of his fingers bent, as if he had been seized with the cramp, and sometimes at his Breast in motion like those of a jockey on full speed; and often he would lift them up as high as he could stretch over his head, for some minutes. But the manoeuvre that used the most particularly to engage the attention of the company was his stretching out his arm with a full cup of tea, in his hand, in every direction, often to the great annoyance of the person who sat next to him, indeed to the imminent danger to their cloaths, perhaps of a Lady’s Court dress; sometimes he would twist himself round with his face close to the back of his chair, and finish his cup of tea, breathing very hard, as if making a laborious effort to accomplish it. (Hill 1897, 273-4)

Present-day commentators on Johnson concur that, judging by the many detailed descriptions of Johnson’s phonic and motor tics, he most probably suffered from Tourette’s syndrome.^4^ The emphasis in these descriptions on Johnson’s obsessive-compulsive behaviour only strengthens the hypothesis, for Tourette’s is frequently accompanied by the symptoms of OCD.^5^ As Beckett’s notes on Johnson acknowledge, the lexicographer never talked about his verbal or motor tics. However, Johnson’s was one of the early dictionaries to include examples of use exclusively from written sources, and as Laura Davies has recently argued, the “Preface” to Johnson’s *Dictionary* makes a clear distinction between speech and writing, associating the former with flux, decay and contingency and the latter with stability, constancy and permanence (2014, 48-52). It is not difficult to see how Johnson’s own inability to control his verbal tics might have resulted in this privileging of the written word. Furthermore, Johnson was suspicious of regional variation in language and, although he accepted the inevitability of linguistic change, he lamented it, noting that “tongues, like governments, have a natural tendency to degeneration,” but that just as “we have long preserved our constitution,” we should “make some struggle for our language” (Johnson 1755-1756, 11).

Beckett’s early interest in dyskinesia and language pathology, in part filtered through his reading of Bergson and his fascination with the cultures of music hall and early cinema, may also be said to account for his life-long interest in Samuel Johnson – an interest that leaves its mark on his own writing. Accounts given by Boswell, Mrs. Thrale and others of Johnson's gesticulations and tics are indeed distinctly Beckettian: the "antics" of Johnson's hands and feet recorded in Hill's book, for instance, find a humorous echo in *Watt* (1953), in the protagonist's idiosyncratic gait:

Watt’s way of advancing due east, for example, was to turn his bust as far as possible towards the north and at the same time to fling out his right leg as far as possible towards the south, and then to turn his bust as far as possible towards the south and then at the same time to fling out his left leg as far as possible towards the north, and then again to turn his bust as far as possible towards the north and to fling out his right leg as far as possible towards the south, and then again to turn his bust as far as possible towards the south and to fling out his left leg as far as possible towards the north, and so on, over and over again, many many times, until he reached his destination, and could sit down. (Beckett 2009c, 23-24)

Motility, as the example from *Watt* so clearly indicates, is rarely transparent in Beckett’s writing. It is frequently stilted through the maiming of the body or permeated with limps, falls and crawls. Movement is frequently presented as unintentional in nature*,* for Beckett’s work contains numerous instances of involuntary movements such as shaking, tics and convulsions. Malone, in the novel *Malone Dies* (1956), for instance, observers that “My arms, once they are in position, can exert a certain force. But I find it hard to guide them. Perhaps the red nucleus has faded. I tremble a little, but only a little” (2010b, 10). Voluntary movements are here compromised, while the involuntary trembling is foregrounded.

Johnson’s habit of manoeuvring his teacup, recorded by Birkbeck Hill, in turn brings to mind a scene from Beckett’s novel *Molloy* (1951), which takes place at the police station after Molloy has been arrested. The protagonist is handed a tray of “greyish concoction which must have been green tea with saccharine and powdered milk” with “a thick slab of dry bread” (2009a, 20). Once the tray is in Molloy’s hands,

The liquid overflowed, the mug rocked with a noise of chattering teeth, not mine, I had none, and the sodden bread sagged more and more. Until, panic-stricken, I flung it all far from me. I did not let it fall, no, but with a convulsive thrust of both my hands I threw it to the ground, where it smashed to smithereens, or against the wall, far from me, with all my strength. (2009a, 21)

This Johnsonian, Tourettic or convulsive aesthetic is everywhere present in Beckett’s writing, extending to the very texture of the language, which is repeatedly staged as speaking itself. The Unnamable, for instance, observes, “I seem to speak, it is not I, about me, it is not about me” (Beckett 2010a, 1). The line anticipates the Unnamable’s female counterpart, Mouth, in Beckett’s 1972 play, *Not I*:

…whole body like gone . . . just the mouth . . . lips . . . cheeks . . . jaws . . . never – . . . what? . . tongue? . . yes . . . lips . . . cheeks . . . jaws . . . tongue . . . never still a second . . . mouth on fire . . . stream of words . . . in her ear . . . practically in her ear . . . not catching the half . . . not the quarter . . . no idea what she’s saying . . . imagine! . . no idea what she’s saying! . . and can’t stop . . . no stopping it . . she who but a moment before . . . but a moment! . . . could not make a sound . . . no sound of any kind . . . now can’t stop . . . imagine! . . can’t stop the stream . . . and the whole brain begging . . . something beginning in the brain . . . beginning the mouth to stop . . . pause a moment . . . if only for a moment . . . and no response . . . as if she hadn’t heard . . . or couldn’t . . . couldn’t pause a second . . . like maddened . . . all that together . . . straining to hear . . . piece it together. . . and the brain . . . raving away on its own . . . trying to make sense of it . . . or make it stop (1986, 380)

Mouth’s monologue is staged as involuntary, convulsive, and beyond intentional control. Its Tourettic logorrhoea takes place in the “nearest lavatory . . . start pouring it out . . . steady stream” (Beckett 1986, 382). The association between enunciation and peristalsis is, in other words, made explicit. In a letter of October 1972 sent to Alan Schneider, the foremost American director of his plays, Beckett writes that he is making a distinction “between mind & voice” in the play and goes on to add that Mouth’s speech is “a purely buccal phenomenon without mental control or understanding, only half heard.” Beckett defines it as “Function running away with organ” (Harmon 1998, 283).

We might conclude that what Beckett responded to in Johnson’s chronic condition was a sense not so much of the unbearable exceptionalism of the lexicographer’s predicament but rather what it unveiled about language and the body in general. In a letter to Mary Manning of 11 July 1937, Beckett responded eloquently and empathically to the dread and misery of Johnson’s unspeakable habits and habitus:

It isn’t Boswell’s wit and wisdom machine that means anything to me, but the miseries that he [Johnson] never talked of, because unwilling or unable to do so. The horror of annihilation, the horror of madness, the horrified love of Mrs. Thrale . . . The opium eating [erasure] dreading-to-go-to-bed, preying-for-the-dead, past living, terrified of dying, terrified of deadness, panting on to 75 bag of water, with a hydrocele on his right testis. How jolly. (Smith 2002, 115)

The letter suggests just how deeply Beckett was influenced by Johnson, but it also suggests that it is the man and his convulsive condition more than the writing that was to leave the most lasting impression on him. There can be little doubt that the body image of the typical Beckett character owes much to Johnson the man – to his wit, intelligence, erudition to be sure, but also, and more pertinently, to his many physical predicaments, including his compulsive verbal and motor tics.

As the Bergsonian notion of the “ready-made” and mechanical nature of language in general recognises, the characteristics of so-called pathological language are also present in non-pathological language use, over 40 % of which is constituted by non-propositional speech acts such as clichés, idioms, and phatic communication. And it is this intrinsic pathology of language that is foregrounded in Beckett’s writing. Beckett’s shift to French in 1946 and the subsequent decision to straddle two languages may itself have been a magical gesture against the “rigid, the ready-made, the mechanical” (Bergson 1998, 127), which Beckett’s writing simultaneously manages to perform, to exemplify and to evade. While Beckett’s writing is far from Cartesian in its stance, it nevertheless explores the Bergsonian notion, informed by medicine and experimental psychology, of the limitations of agency, of “the deep-seated recalcitrance of matter,” and of the human as always already inflicted by the mechanical, a fact that is poignantly highlighted by the case of Samuel Johnson. Through his encounter with Johnson, Beckett registers a paradigm shift in the understanding of subjectivity. Whereas Bergson aims, throughout his career, to *contest* the mechanical, habitual and automatic that threaten to encrust themselves upon the living, in Beckett’s often uncannily Johnsonian writing, the habitual and the automatic become progressively more central, until in the late works, habit and mechanical behavior constitute a tenuous, fraught and primitive ontology, the residues of an agential self.

## Endnotes

^1^ Beckett is also likely to have learned about Bergsonian concepts from Alfred Péron, who was the exchange lecturer at TCD from the École Normale (1926-1928). The character of Chas in *Dream of Fair to Middling Women* (1932/1992) is partly based on Péron. In the novel, Chas talks to a group of students about Bergson: “The difference, then I say, between Bergson and Einstein, the essential difference, is the difference between a philosopher and a sociologist […] And if it is the smart thing nowadays to speak of Bergson as a bit of a cod […] it is that the trend of our modern vulgarity is from the object […] and the idea to sense […] and REASON” (Beckett 1992, 212).

^2^ On 14 October 1930, Beckett wrote to Prentice: “Would you let me add 5 or 6 pages to the last 9? Or would that make it too long? I would like to develop the parallel with Dostoievski and separate Proust’s intuitivism from Bergson’s” (cited in Addyman 2012, 77).

^3^Nordau’s claims so disturbed Émile Zola that he voluntarily underwent a medical examination in order to prove that he did not suffer from neurological disorders.

^4^ See, for instance, Wiltshire 1991, 29-32.

^5^ See, for instance, Towbin 1988.

## References

[CR1] Addyman, David. “’Speak of Time, without Flinching . . .Treat of Space with the Same Easy Grace’: Beckett, Bergson and the Philosophy of Space.” In *Beckett/Philosophy*, edited by Matthew Feldman and Karim Mamdani, 68-88. Sofia: University Press St. Klimrnt Ohridski.

[CR2] Albright D (2003). Beckett and Aesthetics.

[CR3] Beckett, Samuel. 1930s. “*Whoroscope* Notebook.” UoR MS3000. University of Reading Samuel Beckett Collection.

[CR4] ———. 1936-1937. “Human Wishes Notebooks.” UoR MS3461. University of Reading Samuel Beckett Collection.

[CR5] ———. 1986. *Samuel Beckett: The Complete Dramatic Works.* London: Faber.

[CR6] ———. 1992. *Dream of Fair to Middling Women.* Edited by Eoin O’Brien and Edith Fournier. Dublin: Black Cat Press.

[CR7] ———. 1999. *Beckett’s Dream Notebook.* Edited and introduced by John Pilling. Reading: Beckett International Foundation.

[CR8] ———. 2009a. *Molloy.* Edited and introduced by Shane Weller. London: Faber.

[CR9] ———. 2009b. *The Letters of Samuel Beckett 1929-1940.* Edited by Martha Dow Fehsenfeld and Lois More Overbeck. Cambridge: Cambridge University Press.

[CR10] ———. 2009c. *Watt.* Edited and introduced by C. J. Ackerley. London: Faber.

[CR11] ———. 2010a. *The Unnamable.* Edited and introduced by Steven Connor. London: Faber.

[CR12] ———. 2010b. *Malone Dies.* Edited and introduced by Peter Boxall. London: Faber.

[CR13] Bergson, Henri. 1911. *Laughter: An Essay on the Meaning of the Comic*. Translated by Cloudesley Brereton and Fred Rothwell. New York: Macmillan.

[CR14] ———. 1998 *Creative Evolution.* Translated by Arthur Mitchell. New York: Dover.

[CR15] ———. 2001. *Time and Free Will: An Essay on the Immediate Data of Consciousness*. Translated by F. L. Pogson. Mineola: Dover.

[CR16] Billod E (1847). Maladies de la Volonté. Annales Médico-Physiologiques.

[CR17] Boswell, James. 1835. *The Life of Samuel Johnson, LL. D.: Including a Journal of His Tour to the Hebrides; to which Are Added, Anedctodes by Hawkins, Piozzi, Murphy, Tyers, Reynolds, Stevens &c. and Notes by Various Hands.* Vol. V. London: John Murray.

[CR18] ———. 1980. *Life of Johnson*. Edited by R. W. Chapman, introduced by Pat Rogers. Oxford World’s Classics. Oxford: Oxford University Press.

[CR19] Brown, Kate and Howard. L. Kushner. 2001. “Eruptive Voices: Coprolalia, Malediction, and the Poetics of Cursing.” *New Literary History* 32:537-562.

[CR20] Calder J (2001). The Philosophy of Samuel Beckett.

[CR21] Connor, Steven. 2008. “Elan Mortel: Life, Death and Laughter.” Accessed August 5, 2014. http://stevenconnor.com/elanmortel/.

[CR22] Critchley M (1962). Dr. Samuel Johnson’s Aphasia. Medical History.

[CR23] Davies L, Eagle C (2014). Samuel Johnson and the Frailties of Speech. Literature, Speech Disorders, and Disability.

[CR24] Eagle C (2014). Dysfluencies: On Speech Disorders in Modern Literature.

[CR25] Gambor K (2009). Gilded Youth: Three Lives in France’s Belle Époque.

[CR26] Gordon RB (2004). From Charcot to Charlot: Unconscious Imitation and Spectatorship in French Cabaret and Early Cinema. *The Mind of Modernism: Medicine, Psychology, and the Cultural Arts in Europe and America, 1880-1940,* edited and introduced by Mark. S. Micale.

[CR27] Harmon, Maurice (ed). 1998. *No Author Better Served: The Correspondence of Samuel Beckett and Alan Schneider.* Cambridge, MA: Harvard University Press.

[CR28] Harris R (1991). Introduction. In Jean-Martin Charcot’s *Clinical Lecturers on Diseases of the Nervous System*, edited and introduced by Ruth Harris, ix-lxvii.

[CR29] Hill B (1897). *Johnsonian Miscellanies*.

[CR30] Johnson, Samuel. 1755-1756. *A Dictionary of the English Language,* 2 vols. London: J. and P. Knapton et al.

[CR31] ———. *The Letters of Samuel Johnson,* 5 vols. 1992-1994. Edited by Bruce Redford. Oxford: Clarendon Press.

[CR32] Lieberman, Philip. 2000. *Human Language and Our Reptilian Brain: The Subcortical Bases of Speech, Syntax, and Thought.* Cambridge, MA: Harvard University Press. 10.1353/pbm.2001.0011 11253303

[CR33] Maude, Ulrika. 2015. “Beckett, Body and Mind.” In *The New Cambridge Companion to Samuel Beckett,* edited by Dirk Van Hulle, 170-184. Cambridge: Cambridge University Press.

[CR34] Nordau, Max. 1993. *Degeneration*. Introduced by George L. Mosse. Lincoln and London: University of Nebraska Press.

[CR35] Pilling J (1997). Beckett before Godot.

[CR36] Ribot T (1883). Les Maladies de la Volonté.

[CR37] Sacks O (1985). The Man Who Mistook His Wife for a Hat.

[CR38] Schleifer, Ronald. 2001. “The Poetics of Tourette’s Syndrome: Language, Neurobiology, and Poetry.” *New Literary History* 32:563-584.

[CR39] Smith FN (2002). Beckett’s Eighteenth Century.

[CR40] Thrale, Hester Lynch. 1984. *Dr. Johnson and Mrs. Thrale: ‘The Anecdotes’ of Mrs. Piozzi in their Original Form.* Edited by Richard Ingrams. London: Chatto and Windus.

[CR41] Towbin KE, Cohen D, Bruun R, Leckman J (1988). Obsessive-Compulsive Symptoms in Tourette’s Syndrome. Tourette’s Syndrome and Tic Disorders: Clinical Understandings and Treatment.

[CR42] Van Hulle, Dirk and Mark Nixon. 2013. *Samuel Beckett’s Library.* Cambridge: Cambridge University Press.

[CR43] Vulliamy, C. E. 1936. *Mrs. Thrale of Streatham: Her Place in the Life of Dr. Samuel Johnson etc.* London: Jonathan Cape.

[CR44] Wiltshire J (1991). Samuel Johnson in the Medical World.

